# Isolation of bacteria from artificial bronchoalveolar lavage fluid using density gradient centrifugation and their accessibility by Raman spectroscopy

**DOI:** 10.1007/s00216-021-03488-0

**Published:** 2021-07-02

**Authors:** Christina Wichmann, Petra Rösch, Jürgen Popp

**Affiliations:** 1 Leibniz Institute of Photonic Technology Jena – Member of the Research Alliance “Leibniz Health Technologies”, Albert-Einstein-Str. 9, 07745 Jena, Germany; 2 Institute of Physical Chemistry and Abbe Center of Photonics, Friedrich Schiller University Jena, Helmholtzweg 4, 07743 Jena, Germany; 3 Research Campus Infectognostics, Philosophenweg 7, 07743 Jena, Germany

**Keywords:** Raman spectroscopy, Bacteria, Isolation

## Abstract

Raman spectroscopy is an analytical method to identify medical samples of bacteria. Because Raman spectroscopy detects the biochemical properties of a cell, there are many factors that can influence and modify the Raman spectra of bacteria. One possible influence is a proper method for isolation of the bacteria. Medical samples in particular never occur in purified form, so a Raman-compatible isolation method is needed which does not affect the bacteria and thus the resulting spectra. In this study, we present a Raman-compatible method for isolation of bacteria from bronchoalveolar lavage (BAL) fluid using density gradient centrifugation. In addition to measuring the bacteria from a patient sample, the yield and the spectral influence of the isolation on the bacteria were investigated. Bacteria isolated from BAL fluid show additional peaks in comparison to pure culture bacteria, which can be attributed to components in the BAL sample. The isolation gradient itself has no effect on the spectra, and with a yield of 63% and 78%, the method is suitable for isolation of low concentrations of bacteria from a complex matrix.

**Supplementary Information:**

The online version contains supplementary material available at 10.1007/s00216-021-03488-0.

**Figure:**
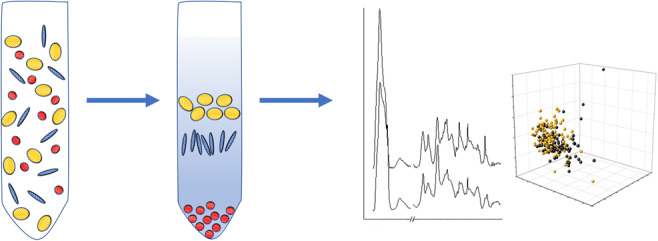
Graphical abstract

## Introduction

The identification of bacteria is of great importance in research and medicine. The current gold standard of cultivation has increasingly evolved toward analytical methods that reduce time, cost and effort, such as Raman spectroscopy. Raman spectroscopy is a vibrational spectroscopic method, so it detects many adaptations of the cell to the changing environment, and spectra can be influenced by many parameters [1]. Therefore, a number of aspects have to be considered before starting the experiment, to avoid influencing the spectra. These possible sources of alteration can be divided into three main groups: device-related, sample-related and experiment-related.

Before starting an experiment, among other things, an appropriate laser wavelength [2] needs to be selected, and laser power and integration time need to be adjusted according to the sample to achieve a balance that provides enough power to increase the signal while also not burning the sample. Furthermore, the handling of the sample needs to be discussed, since different sample substrates might interfere with the sample spectra [3, 4]; obviously one wants to avoid peaks of the substrate interfering with the fingerprint region of the sample and thus hampering the analysis of the measured data.

For biological samples like bacteria, many factors can change the spectra. Even in pure cultures, many parameters regarding their cultivation can influence the spectra and therefore the characterization of the bacterial species. One important consideration is the choice of medium and cultivation method. Some media or media supplements cause their own Raman signal, which needs to be avoided [5–7]. Before starting cultivation, one must be aware of the differences between liquid and solid media [5]. During shaking, the bacteria are constantly exposed to new media and sources of nutrition [5]. The same medium in liquid or solid form can result in different phenotypes of the same bacterial species [5]. This can have the effect that some bacteria can be distinguished spectroscopically much better after cultivation in liquid medium than after cultivation on solid medium [8].

After all conditions of cultivation are considered, the next step is to define the time when samples are taken for analysis, since the age or growth phase of the culture also affects biological variations in the bacteria and thus in the spectra [9, 10]. Culture conditions such as the concentration of CO_2_ during growth can also influence the metabolism and consequently the spectra [11]. Therefore, it is possible to observe the change from aerobic to microaerophilic metabolism in *Escherichia coli* by Raman spectra [12], and low salt concentrations in the medium are depicted by changes in amino acids and fatty acids [13].

For the experiment-related influences, the organization of lab and experimental times needs to be considered. Different storage conditions of the bacteria samples before measurement can cause bacteria to experience stress and produce different effects on the spectra [14]. The same applies to the component of time between preparation and measurement or centrifugation, and its influence on different spectra should not be underestimated [15].

While there are already numerous influences on Raman spectra of bacteria in pure cultures, the influence is even more evident in environmental or patient samples, as many parameters in the environment cannot be controlled externally. In medical or environmental samples, the bacteria are usually embedded in a matrix of some kind, which has to be removed from single cells before examination. Therefore, sample preparation before measurement should not further harm the bacteria. Especially for the examination of single cells, an adapted isolation is needed which does not modify the bacterial surface or stress the bacteria unnecessarily [16]. In classical microbiological experiments, filtration [17] or homogenization of the matrix [18] followed by cultivation on selective agar [17, 18] has been used. Introducing a cultivation step into the isolation strategy might influence the bacteria phenotype and therefore might negatively affect the Raman spectroscopic identification potential.

For the isolation, it is necessary to adapt the method to the subsequent analysis technique; for example, for Raman spectroscopy, it needs to be nondestructive. Of course, there is a wide range of possible isolation techniques with different levels of complexity. Simple isolation methods range from culture-independent filtration [19] and simple centrifugation [20] to a combination of both methods. In more complex matrices, it might be necessary to chemically digest the surrounding matrix until no residues are adhered to the bacteria which might interfere with the Raman spectrum. This approach was shown, for example, for the isolation of bacteria from blood or sputum, which in the end showed that there was no longer an influence of hemoglobin or mucus in the bacterial spectra [6, 21]. Surface-modified nanoparticles can also be used, which bind to the bacteria [22], and this connection is then isolated from the matrix by, for example, a magnet [23]. In dielectrophoresis, bacteria are trapped in a certain area by an electric field to enrich the concentration of particles. With this method it is possible to enrich bacteria from urine at one point and record Raman spectra there [24]. A similar approach is used with optical tweezers, where cells are not concentrated on a point by an electric field but by a focused laser beams and can be measured there [25].

Therefore, although it is a great challenge, it is possible to isolate bacteria from complex matrices using Raman-compatible methods. In this contribution, we present a method to isolate bacteria from bronchoalveolar lavage (BAL) fluid using density gradient centrifugation. The BAL sample was taken from a healthy volunteer, without knowledge of further details regarding the composition of the sample. For bacterial isolation, the density gradient centrifugation method was chosen, in which a sample is separated into its individual components by their density. The isolation of bacteria from BAL fluid is complicated because of its complex matrix. When a BAL sample is taken, the lung surface is rinsed with sterile NaCl solution, which is why it contains the entire biology of the lung including mucus, microbes such as bacteria, fungi or viruses, and sometimes even blood. Moreover, there are also very few bacteria in a BAL sample, and this amount also varies between individual patients. On average, BAL fluid contains 7 × 10^4^ cells/ml [26, 27], but samples with as few as 1000 cells/ml have been reported [28].

## Material and methods

### Density gradient and centrifugation parameters

The principle of density gradient centrifugation is based on separating the individual components of a complex sample through a separation medium according to the densities of the individual components. The separation medium is pipetted in such a way that the density at the lower end of the vessel increases. During centrifugation, the sample is centrifuged through the gradient, and the individual particles remain at the gradient density level that matches their own density. Frequently used solutions are Percoll [29], Ficoll [30], sucrose [31] and OptiPrep [32], the last of which was used in this study. Therefore, two different solutions were used and mixed together. The first solution was OptiPrep (Sigma-Aldrich), with a density of 1.319 g/ml. The second solution was an in-house-made Galantine buffered solution (GBS) consisting of 0.85 g NaCl, 0.03 g KH_2_PO_4_ and 0.01 g galantine (Sigma-Aldrich) dissolved in 100 ml distilled water. GBS was used to dilute OptiPrep to the desired concentrations of 35%, 30%, 25% and 20%. The calculated densities of the gradient were 1.1066 g/ml, 1.0903 g/ml, 1.0631 g/ml and 1.0576 g/ml, respectively, which had already been shown to be suitable for isolation of bacteria [29]. To pipette the gradient, a 15 ml Falcon tube was used because of the narrow tip, and 500 μl of each of the different densities of the gradient was pipetted in descending concentration. The sample was added on top of the gradient and centrifuged in a swinging-bucket rotor (2700×*g* for 60 min at 23 °C).

### Density gradient centrifugation to isolate bacteria from a BAL sample

BAL fluid consists of the entire biology of the lung surface, including not only bacteria and other microorganisms, but also blood, mucus, epithelial cells and other components of the immune system. Therefore, the sample needs to be purified before bacteria can undergo further investigation such as Raman spectroscopy. To test the accessibility of the gradient for medical samples, a BAL sample from a healthy volunteer was used. Approval was obtained from the volunteer and the independent Ethics Committee of the University of Lübeck (16-145). For the BAL sample, five aliquots of 1 ml were taken in 0.9% NaCl, and because BAL fluid contains only a few bacteria, all 5 ml were combined and added to the gradient. Then all tubes were washed with the same 1 ml water to collect all bacteria which might still be attached to the containers, thus minimizing sample loss at the surface of the tubes. After the isolation, the supernatant was removed, and the pellet was suspended in 1 ml H_2_O and centrifuged in a fixed-angle rotor for 5 min at 10,000×*g* and 23 °C before the water was removed and the washing step repeated a further two times. Small droplets of the sample were placed on a nickel slide and dried at room temperature before Raman measurements were performed. In all, 531 particles were measured in this sample.

**Fig. 1:**
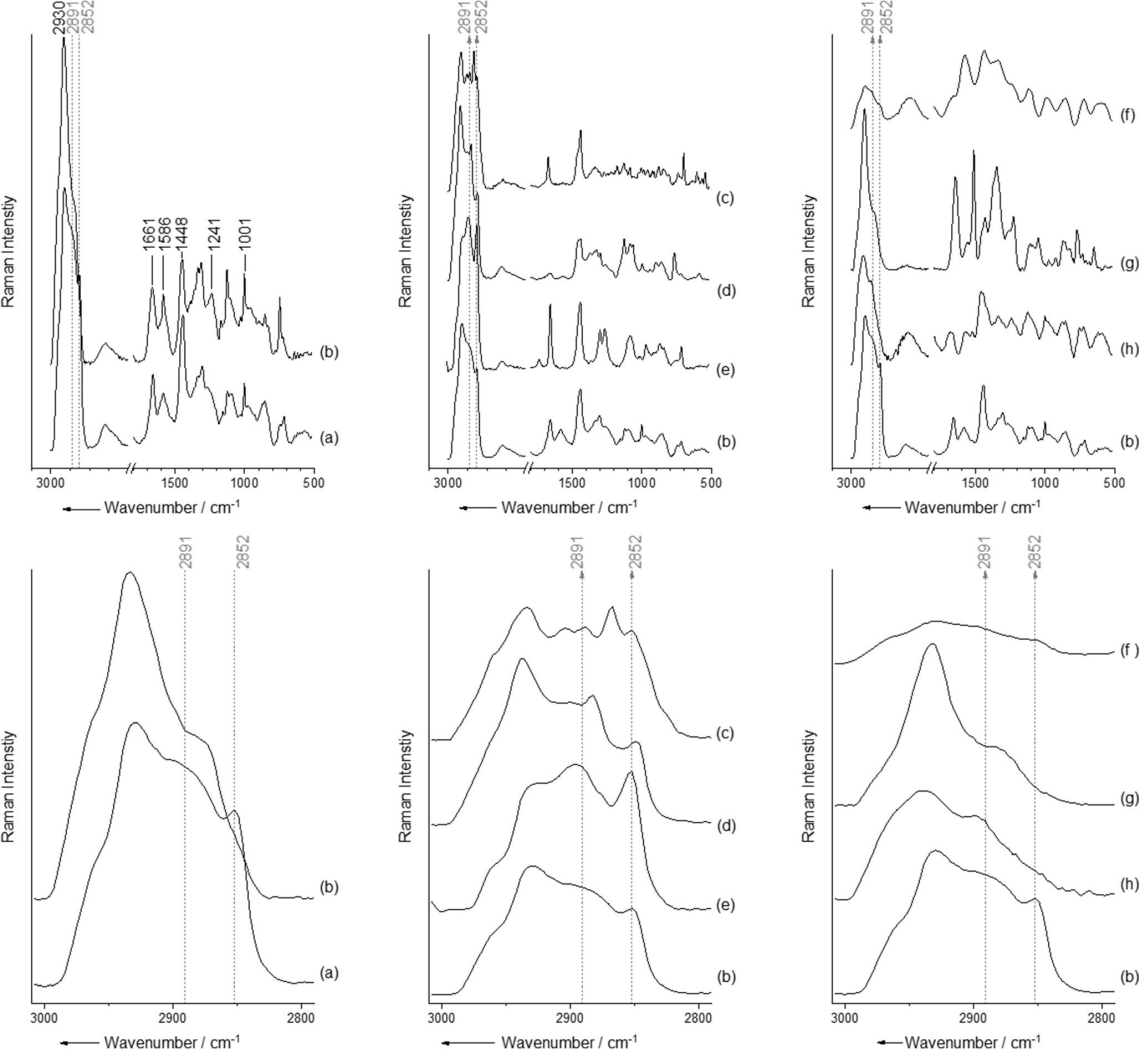
Influence of density gradient centrifugation on bacterial Raman spectra. The upper row shows the entire Raman spectrum; the lower row shows a magnification of the ν(C-H) signal. Dotted lines are drawn at 2891 cm^−1^ and 2852 cm^−1^. Comparison of the mean spectra of pure culture of *S. thermophilus* after density gradient centrifugation (**a**) and mean spectra of all 531 particles isolated from BAL fluid (**b**). Additional lipid peaks in BAL fluid (**b**) in comparison to pure culture (**a**) can be mainly assigned to cholesterol (c), lipopolysaccharides (d) and phospholipids (e). Background of the BAL (f), OptiPrep (g) and artificial sputum (h) shows a minor influence on the mean spectra. For better visualization, the mean spectra of bacteria isolated from BAL fluid are shown in all three panels (b)

To investigate the influence of the matrix or the presence of other biological particles in the sample on the Raman spectra, a pure culture of *Streptococcus thermophilus* was obtained in parallel as a negative control and was measured. All recorded spectra were passed to the data evaluation without previous sorting. The mean spectrum of all measured particles from the BAL sample depicted a bacterial spectrum, but some additional signals appeared (see Fig. 1), which might have originated in the OptiPrep or sputum in the BAL fluid.

To determine the source of any additional peaks, a culture of bacteria was compared to bacteria isolated from an artificial BAL sample as well as the artificial sputum and some of its single components, namely phospholipids, lipopolysaccharides and cholesterol. The background of BAL fluid, i.e. non-bacterial particles, was also included. Artificial sputum was prepared as described previously [21]. For bacterial control samples, *S. thermophilus* DSM 20617 (Leibniz Institute–German Collection of Microorganisms and Cell Cultures) was used. The culture of *S. thermophilus* was suspended in 0.9% NaCl with an OD_600_ of 0.3 before it was diluted 1:2 in water. One hundred microliters of this bacterial solution was mixed with 900 μl artificial sputum and 5 ml 0.9% NaCl to simulate a patient BAL sample with low bacterial load. Simulated BAL fluid was subjected to density gradient centrifugation before measurement, while the artificial sputum and components were measured as pure components.

### Influence of the gradient on bacterial Raman spectra and yield of the isolation

To determine the influence of the gradient on bacterial spectra, a pure culture of *S. thermophilus* without sputum was used to confirm that the influence originated from the gradient and not from the sputum. Bacteria were cultivated on brain-heart infusion (BHI) agar overnight and suspended in sterile distilled water before they were divided into two parts. The first part was prepared for Raman measurement without gradient, while the other part was treated by density gradient centrifugation. The first part served as a negative control, to verify that the spectral changes in the second group were due to centrifugation and the density gradient.

Another goal of isolation is a good yield. Since it is not known specifically which or how many bacteria are present in a BAL sample, a culture of *S. thermophilus* was used. *S. thermophilus* was diluted in 0.9% NaCl to an optical density at 600 nm wavelength (OD_600_) of 0.09 before it was diluted with H_2_O at a ratio of 1:10. Five microliters of this solution was added to BHI agar in triplicate, and the remainder of the solution was used for density gradient centrifugation. After the gradient supernatant was removed, the bacteria pellet was dissolved in 1 ml H_2_O, from which 5 μl was plated on BHI. After incubation at 37 °C for 24 h, the number of colony-forming units (CFU) was evaluated and compared to the number of bacteria before the gradient in three independent runs.

### Spectroscopic investigation and data analysis

Raman spectroscopic investigations were performed using a commercially available Raman platform (Bio Particle Explorer, rap.ID, Berlin, Germany) equipped with a frequency-doubled (532 nm), solid-state diode-pumped Nd:YAG laser (LCM-S-11-NNP25, Laser-export Co. Ltd., Moscow, Russia). The laser beam is focused by an Olympus MPL-LFN-BD 100× objective (Olympus Corporation, Tokyo, Japan) onto the sample with a spot size of less than 1 μm and maximum laser intensity of approximately 10 mW. The backscattered Raman light is diffracted by a single-stage monochromator with a 920 line/mm grating (HE532, Horiba Jobin Yvon, Munich; Germany), before its detection with a thermoelectrically cooled CCD (DV 401_BV; Andor Technology). Thus, a spectral resolution of approximately 10 cm^−1^ is provided. Before measurement of the bacterial samples, 4-acetamidophenol (4-AAP) was measured for the calibration of the *x*-axis. Bacteria were irradiated with a laser power of 50% corresponding to approximately 5 mW for 5 s. In order to determine whether the isolation technique was suitable for Raman measurements, the mean spectra of the data were calculated. For this step, raw spectra need to be cleaned from non-sample-related signals such as cosmic rays, fluorescence or spectral artifacts. Data preprocessing was done using in-house-designed software for Raman data analysis [33]. The initial step of the data analysis was the removal of cosmic spikes [34] and the calibration of the wavenumbers with the spectra of 4-AAP. To eliminate the fluorescent background, the baseline was adjusted using a sensitive nonlinear iterative peak (SNIP) clipping algorithm. The last step was cropping the silent region from 1800 to 2600 cm^−1^ and vector normalization before the preprocessed mean spectra were calculated. To compare pure culture spectra before and after density gradient centrifugation, a combination of principal component analysis (for dimension reduction) and supervised linear discriminant analysis (PCA-LDA) was performed. Seven principal components and an inner evaluation of tenfold cross-validation were used. With this combination of chemometric methods, the data are reduced in dimension by PCA and separated by maximal diversity by LDA.

## Results and discussion

Environmental or medical bacterial samples may be embedded in complex matrices, which hamper Raman spectroscopic identification. Therefore, it is important to purify the samples before measurement. Pre-cultivation is not always possible, as it is not known which bacteria are present, especially in medical samples. In addition, generally only a fraction of these bacteria can be cultivated, which makes cultivation and enrichment almost impossible in these cases. Therefore, the bacteria must be isolated from the sample. An appropriate isolation method fulfills two main goals. One goal is a good yield, where as many bacteria as possible can be investigated. This is especially relevant for samples which do not contain many bacteria, such as BAL fluid. The second goal is to use an isolation which is suitable for further methods of investigation. For Raman spectroscopic analysis of BAL fluid, both goals are of utmost importance, since BAL samples contain very few bacteria, and the isolation method needs to be nondestructive so that a phenotypic method like Raman measurement is possible.

### Accessibility to clinical samples

To test whether the isolation method presented here is suitable for BAL fluid, a sample was obtained from a volunteer, and the bacteria were isolated by density gradient centrifugation and measured using Raman spectroscopy. As for all medical samples, bacteria in BAL samples differ in their morphology when compared to pure culture and might appear smaller. A total of 531 particles were measured, and all spectra were calculated to a mean value spectrum, regardless of whether bacteria or other particles were measured. Although BAL fluid is neither a clean sample containing only bacteria nor a pure culture, the mean spectrum of this measurement depicts a bacterial Raman spectrum (Fig. 1). A typical bacterial Raman spectrum consists of the peaks representing ν(C-H) at 2930 cm^−1^, amide I at 1661 cm^−1^, DNA at 1586 cm^−1^, δ(CH_2_/CH_3_) at 1448 cm^−1^, amide III at 1241 cm^−1^ and phenylalanine at 1001 cm^−1^ [1].

Compared to spectra of a bacterial culture, some peaks appear to be different in the spectrum of bacteria isolated from BAL fluid (spectra a and b in Fig. 1). Signals representing ν(C-H), amide I, δ(CH_2_/CH_3_) and phenylalanine are clearly visible, while the signals of DNA and amide III are less prominent. Since bacteria in medical samples are smaller than cultivated cells, they may have lower DNA concentrations. Also, changes in DNA and amide peaks in the spectra can be observed after transport and storage of samples [14]. In comparison to the spectra of pure cultures (Fig. 1a), some additional signals appear. Additional shoulders appear at 2891 cm^−1^ and 2852 cm^−1^, representing lipids. To find the origin of these additional signals, the different components of the artificial sputum were measured, and pure cultures of bacteria were added to sputum and NaCl to check whether small traces of sputum remained attached to the bacteria and therefore affect the spectrum. In the end, the peaks at 2891 cm^−1^ and 2852 cm^−1^ were determined to originate from phospholipids, lipopolysaccharides and cholesterol (Fig. 1). Phospholipids in particular are found to be part of sputum [35] and may adhere to the surface of bacteria and therefore interfere with the resulting Raman spectrum.

### Quality control of the isolation method

**Fig. 2:**
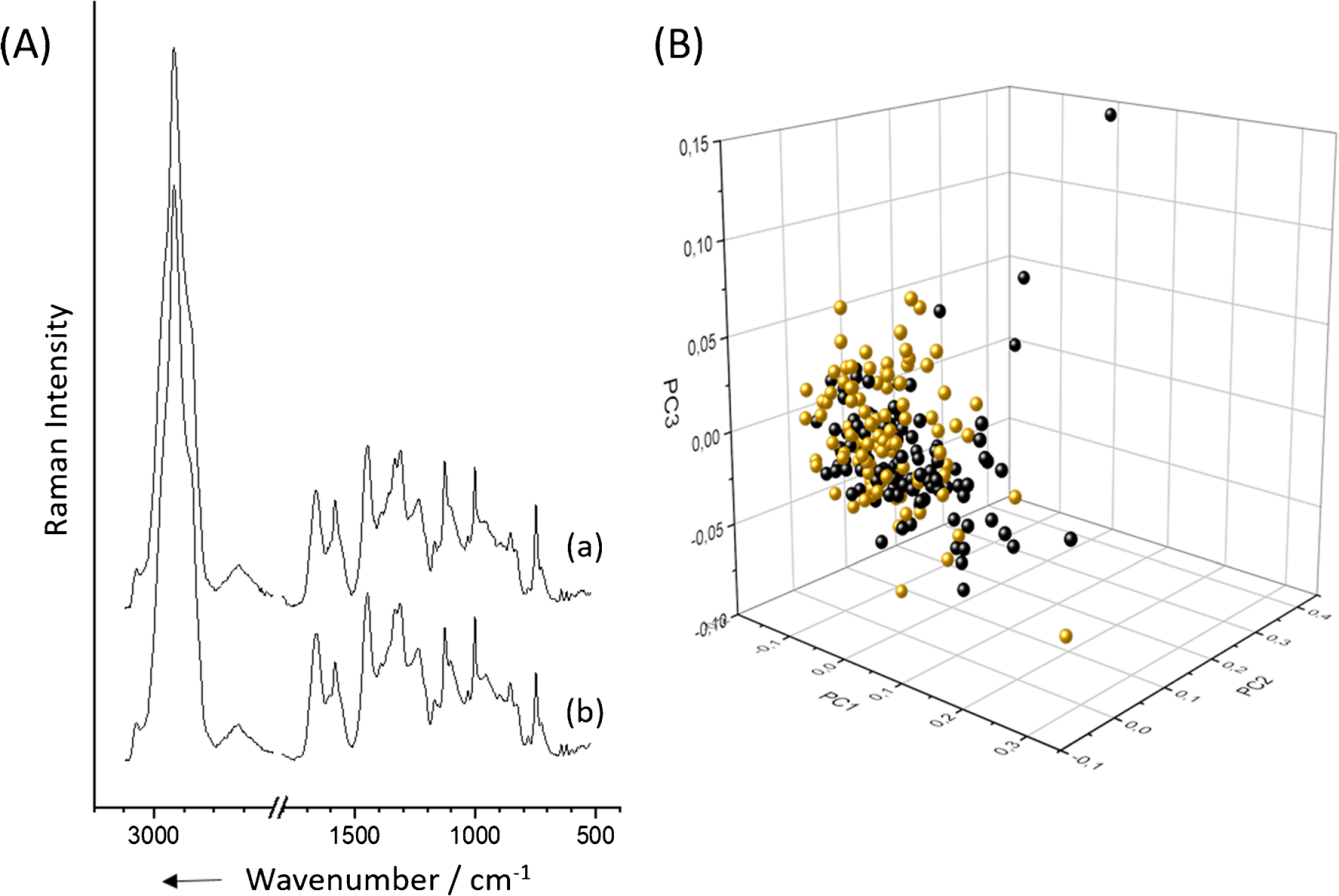
Influence of density gradient centrifugation on bacterial Raman spectra. **A** Mean spectra of *S. thermophilus* in pure culture (b) and after density gradient centrifugation (a) as the average of each 100 single-cell Raman spectra. **B** PCA-LDA score plots for the classification of the same bacterial Raman spectra before (yellow) and after (black) density gradient centrifugation

Having established that it is possible to isolate the bacteria of a BAL sample by density gradient centrifugation, the influence of the gradient and the yield were investigated. In order to investigate the influence of the gradient on the spectra, a pure culture of bacteria was used to ensure that possible influences really come from the gradient and not from artificial sputum or other components of the BAL fluid. Possible influences were examined by comparing the mean spectra and applying PCA-LDA to separate the data into two groups. The results are shown in Fig. 2, and did not identify any differences (see also Figs. S1 and S2 in Electronic Supplementary Information, ESM). The mean spectra look the same, and chemometric analysis was not able to distinguish between groups.

Another important feature of a newly introduced isolation method is the determination of the yield. Since an appropriate isolation method should obviously lose as few bacteria as possible, we calculated the yield of our isolation by plating. Therefore, we used the same bacterial solution in three independent gradient centrifugation runs and plated 5 μl of each centrifugation sample on each three independent plates before and after isolation and calculated the yield (see Table 1). The number of bacteria in this test run was 5.7 × 10^3^ CFU/ml, below the average bacterial load of a BAL sample of 7 × 10^4^ CFU/ml [26, 27]. Therefore, if an acceptable yield is possible with such a low bacterial load, this method can also be used for samples with higher concentrations.

**Table 1:** Yield of isolation by density gradient centrifugation of a pure bacterial culture after overnight incubation of the bacterial suspension on BHI agar. Incubation of the stock solution yielded 5.7 × 10^3^ CFU/ml

	Absolute number of CFU/ml			Relative yield of CFU/ml		
	Sample 1	Sample 2	Sample 3	Sample 1	Sample 2	Sample 3
Mean	3.6 × 10^3^	4.3 × 10^3^	4.5 × 10^3^	63.1%	75.4%	78.9%

For this density gradient, an isolation rate of between 63% and 78% was achieved. On the first sight the yields might seem low but they are comparable with other isolation methods from complex matrices. A study about bacteria in whole blood used an electrical field applied to the sample to concentrate the bacteria in one spot and record spectra there with a yield between 40 and 50% [36]. Lorenz et al. isolated bacteria from blood and achieved a yield of about 70%. They explained that bacteria could possibly adhere to the surfaces of pipette tips and reaction vessels, which could explain the loss during isolation [6]. For the isolation of bacteria from milk, yields of between 8% and 53% were achieved with various isolation methods [29]. In both studies, lower yields were observed in less concentrated bacterial solutions. Optical tweezers can also be used for isolation of bacteria. One study reported that 61% of yeasts and 38% of bacteria were successfully isolated from a mixed culture of yeasts and bacteria using this technique [37]. Therefore, the yield achieved with our isolation method can be regarded as satisfactory.

## Conclusion

In this study, we addressed the problem of how to isolate bacteria from a complex matrix with a low microbial load using density gradient centrifugation. For this purpose, a BAL sample from a healthy volunteer was taken and isolated by density gradient centrifugation. More than 500 particles were measured and showed bacterial Raman signals. Thus, we can assume that the introduced density gradient centrifugation is suitable for nondestructive isolation of bacteria from BAL samples so that Raman measurements can be performed. For quality control of the isolation technique, the yield of the isolation method was evaluated using a pure culture of *S. thermophilus*, which resulted in a recovery of between 63% and 78%. Since the exact bacterial composition of a BAL sample is unknown, it is not possible to calculate the efficiency of the isolation technique. In this case, only a qualitative evaluation was done to determine whether the bacteria could be isolated. No influence of residues of the isolation gradient was observed.

## Supplementary Information

ESM 1 (PDF 241 kb)

## Declarations

The sample was taken from a volunteer in Leibniz Lung Center and Clinics in Borstel. Approval was obtained from the volunteer and the independent Ethics Committee of University Lübeck (16-145). No violation of human or animal rights occurred during this investigation.
