# Supplementary material for: Isolation of bacteria from artificial bronchoalveolar lavage fluid using density gradient centrifugation and their accessibility by Raman spectroscopy
Source: Anal Bioanal Chem. 2021 Jul 2;413(20):5193–200. doi: 10.1007/s00216-021-03488-0 (PMC8405473; doi:10.1007/s00216-021-03488-0)
Supplement: Supplementary file 1 — (PDF 241 kb) [file 216_2021_3488_MOESM1_ESM.pdf]

Supporting Information

Isolation of bacteria from artificial bronchoalveolar lavage fluid using density gradient centrifugation and their accessibility by Raman spectroscopy

Christina Wichmann[1,2,3], Petra Rösch[2,3,*], Jürgen Popp[1,2,3]

1) Leibniz Institute of Photonic Technology Jena – Member of the research alliance "Leibniz Health Technologies", Albert-Einstein-Str. 9, 07745 Jena, Germany

2) Institute of Physical Chemistry and Abbe Center of Photonics, Friedrich Schiller University Jena, Helmholtzweg 4, 07743 Jena, Germany

3) Research Campus Infectognostics, Philosophenweg 7, 07743 Jena, Germany

** corresponding author Dr. Petra Rösch: petra.roesch@uni-jena.de*

Supplement

Table of Content

PCA variance plot for selecting the number of PCs S2

2D plot for classification of bacterial Raman spectra before and after isolation................................. S3

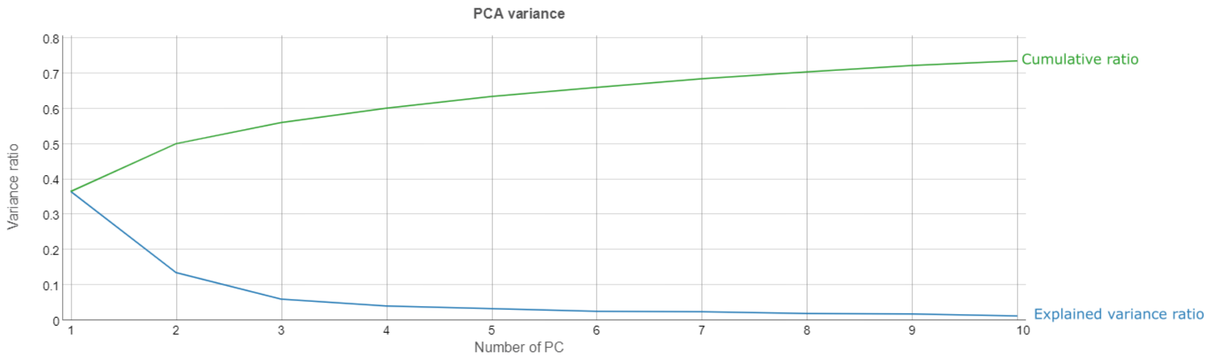

Figure S1: PCA variance plot for selecting the number of PCs used for the comparison of Raman spectra of bacteria before and after density gradient centrifugation to investigate influences of the gradient to the bacterial spectra.

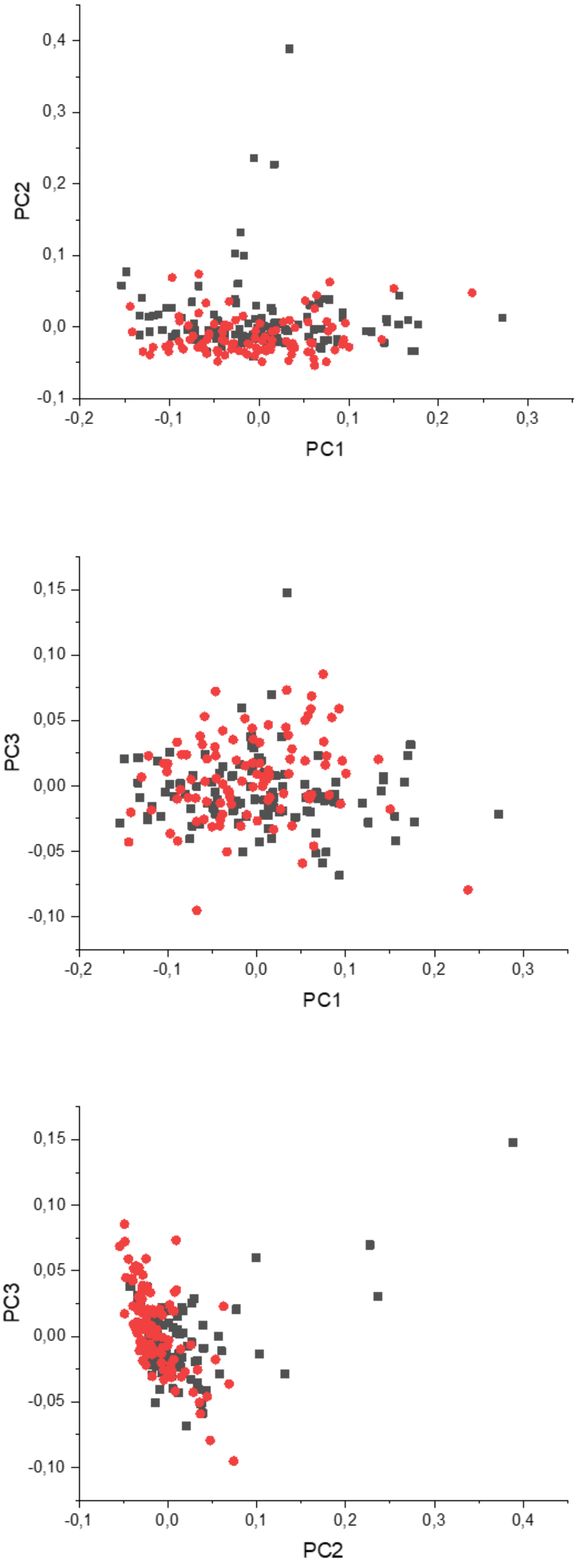

Figure S2: 2D plot of PC 1-3 for the classification of the same bacterial Raman spectra before (red) and after (black) the density gradient centrifugation to investigate the influence of density gradient centrifugation to bacterial Raman spectra
